# Re-thinking the Etiological Framework of Neurodegeneration

**DOI:** 10.3389/fnins.2019.00728

**Published:** 2019-07-24

**Authors:** Ximena Castillo, Susana Castro-Obregón, Benjamin Gutiérrez-Becker, Gabriel Gutiérrez-Ospina, Nikolaos Karalis, Ahmed A. Khalil, José Sócrates Lopez-Noguerola, Liliana Lozano Rodríguez, Eduardo Martínez-Martínez, Claudia Perez-Cruz, Judith Pérez-Velázquez, Ana Luisa Piña, Karla Rubio, Héctor Pedro Salazar García, Tauqeerunnisa Syeda, America Vanoye-Carlo, Arno Villringer, Katarzyna Winek, Marietta Zille

**Affiliations:** ^1^Instituto de Neurobiología, Universidad Nacional Autónoma de México, Mexico City, Mexico; ^2^Institute of Neurobiology, University of Puerto Rico, San Juan, PR, United States; ^3^Instituto de Fisiología Celular, Universidad Nacional Autónoma de México, Mexico City, Mexico; ^4^Artificial Intelligence in Medical Imaging KJP, Ludwig Maximilian University of Munich, Munich, Germany; ^5^Laboratorio de Biología de Sistemas, Departamento de Biología Celular y Fisiología, Instituto de Investigaciones Biomédicas y Coordinación de Psicobiología y Neurociencias, Facultad de Psicología, Universidad Nacional Autónoma de México, Mexico City, Mexico; ^6^Friedrich Miescher Institute for Biomedical Research, Basel, Switzerland; ^7^Center for Stroke Research Berlin, Charité-Universitätsmedizin Berlin, Berlin, Germany; ^8^Berlin School of Mind and Brain, Humboldt-Universität zu Berlin, Berlin, Germany; ^9^Department of Neurology, Max Planck Institute for Human Cognitive and Brain Sciences, Leipzig, Germany; ^10^School of Health Sciences, Department of Gerontology, Autonomous University of Hidalgo State, Pachuca, Mexico; ^11^Departamento de Bioquímica, Facultad de Medicina, Universidad Nacional Autónoma de México, Mexico City, Mexico; ^12^Cell Communication & Extracellular Vesicles Laboratory, Instituto Nacional de Medicina Genómica, Mexico City, Mexico; ^13^National Polytechnic Institute, Center of Research in Advanced Studies, Mexico City, Mexico; ^14^Departamento de Matemáticas y Mecánica, Instituto de Investigaciones en Matemáticas Aplicadas y Sistemas, Universidad Nacional Autónoma de México, Mexico City, Mexico; ^15^Mathematische Modellierung Biologischer Systeme, Fakultät für Mathematik, Technische Universität München, Munich, Germany; ^16^Department of Neurosurgery, Charité-Universitätsmedizin Berlin, Berlin, Germany; ^17^Lung Cancer Epigenetics, Max Planck Institute for Heart and Lung Research, Bad Nauheim, Germany; ^18^Leibniz-Forschungsinstitut für Molekulare Pharmakologie, Berlin, Germany; ^19^Laboratorio de Neurociencias, Instituto Nacional de Pediatría, Secretaría de Salud, Mexico City, Mexico; ^20^The Shimon Peres Postdoctoral Fellow at the Edmond and Lily Safra Center for Brain Sciences, The Hebrew University of Jerusalem, Jerusalem, Israel; ^21^Department of Experimental Neurology, Charité-Universitätsmedizin Berlin, Berlin, Germany; ^22^Institute for Experimental and Clinical Pharmacology and Toxicology, University of Lübeck, Lübeck, Germany; ^23^Institute for Medical and Marine Biotechnology, University of Lübeck, Lübeck, Germany; ^24^Fraunhofer Research Institution for Marine Biotechnology and Cell Technology, Lübeck, Germany

**Keywords:** lifespan, vascular pathology, senescence, body–brain trophism, dysbiosis

## Abstract

Neurodegenerative diseases are among the leading causes of disability and death worldwide. The disease-related socioeconomic burden is expected to increase with the steadily increasing life expectancy. In spite of decades of clinical and basic research, most strategies designed to manage degenerative brain diseases are palliative. This is not surprising as neurodegeneration progresses “silently” for decades before symptoms are noticed. Importantly, conceptual models with heuristic value used to study neurodegeneration have been constructed retrospectively, based on signs and symptoms already present in affected patients; a circumstance that may confound causes and consequences. Hence, innovative, paradigm-shifting views of the etiology of these diseases are necessary to enable their timely prevention and treatment. Here, we outline four alternative views, not mutually exclusive, on different etiological paths toward neurodegeneration. First, we propose neurodegeneration as being a secondary outcome of a primary cardiovascular cause with vascular pathology disrupting the vital homeostatic interactions between the vasculature and the brain, resulting in cognitive impairment, dementia, and cerebrovascular events such as stroke. Second, we suggest that the persistence of senescent cells in neuronal circuits may favor, together with systemic metabolic diseases, neurodegeneration to occur. Third, we argue that neurodegeneration may start in response to altered body and brain trophic interactions established via the hardwire that connects peripheral targets with central neuronal structures or by means of extracellular vesicle (EV)-mediated communication. Lastly, we elaborate on how lifespan body dysbiosis may be linked to the origin of neurodegeneration. We highlight the existence of bacterial products that modulate the gut-brain axis causing neuroinflammation and neuronal dysfunction. As a concluding section, we end by recommending research avenues to investigate these etiological paths in the future. We think that this requires an integrated, interdisciplinary conceptual research approach based on the investigation of the multimodal aspects of physiology and pathophysiology. It involves utilizing proper conceptual models, experimental animal units, and identifying currently unused opportunities derived from human data. Overall, the proposed etiological paths and experimental recommendations will be important guidelines for future cross-discipline research to overcome the translational roadblock and to develop causative treatments for neurodegenerative diseases.

## Introduction

Neurological disorders are the leading cause of disability-adjusted life-years and the second-leading cause of deaths worldwide. This burden has increased substantially over the past 25 years because of expanding population numbers and aging (GBD 2015 Neurological Disorders Collaborator Group, 2017) and will continue to grow in the coming decades due to the further increase in life expectancy.

At intermediate and advanced stages, frequently by the time a diagnosis is made, neurodegeneration has affected higher brain areas that control cognitive, sensory, and motor functions. Because the majority of cases are seen by the age of 50 or older in both men and women, many scientists think of neurodegenerative diseases as processes that reflect abnormal aging. Likewise, since neurodegeneration involves the loss of neuronal populations brain-wide, many researchers believe that neuronal death results from altered processes intrinsic to the nervous system, such as neuroinflammation, abnormal accumulation or deteriorated clearance of toxic proteins, and reduced anti-oxidative defenses.

Hence, current explicative models of neurodegenerative diseases account for signs and symptoms already seen in affected patients. In fact, the majority of these causative models have been formulated in retrospect, based on what is already present in the patient’s brain. Given the nature of these models, it is difficult to identify real causes from mere consequences and epiphenomena. This is even more puzzling given that neurodegenerative diseases are life-spanning processes that start silently at least 20 to 30 years before the pathological signs and symptoms are recognized by patients and diagnosed by physicians. In addition, accumulating information suggests an extraneural origin of neurodegeneration.

Clearly, the essential premises guiding research on neurodegenerative diseases seem at least partly incorrect and somewhat misleading. This may explain why, despite researchers’ best efforts, neurodegeneration is still a growing health concern and the reason why therapeutic measures are currently palliative, while preventive approaches are often ineffective (Crous-Bou et al., 2017; Hsu and Marshall, 2017; Klein and Tyrlikova, 2017).

Here, we outline four different paths as the basis for a paradigm shift toward an etiologic view on neurodegeneration: (1) the vascular origin of neurodegeneration, (2) cellular senescence as the origin of neurodegeneration, (3) body–brain trophic interactions as the origin of neurodegeneration, and (4) lifespan gut dysbiosis as the origin of neurodegeneration. We did not cover here genetic components as risk factors for neurodegeneration, a comprehensive analysis of which can be found elsewhere (Brainstorm et al., 2018). We provide concrete recommendations on how to investigate the discussed etiological paths experimentally in future studies. This work is the result of the discussions that took place during the German-Mexican Roundtable Exploring New Etiological Paths Towards Neurodegeneration in October 2018.

## The Vascular Origin of Neurodegeneration

Neurons depend on blood vessels to deliver oxygen and nutrients, for the removal of carbon dioxide and other by-products of metabolism from the brain’s interstitial space, which helps to maintain the homeostasis of the cerebral microenvironment. Therefore, to function properly, it is thought that the cerebral circulation has developed adaptive mechanisms to provide the trophic molecules and energy substrates needed for neuronal cells at different times and regions, depending on neural activity (Girouard and Iadecola, 2006).

The recognition of these mechanisms led to the concept of neurovascular coupling (Roy and Sherrington, 1890), which refers to the dynamic functional change in cerebral blood flow that occurs in response to local neuronal activity (Dirnagl, 1997; Kisler et al., 2017; Villringer, 1997). This response increases cerebral blood flow within the activated brain regions to fulfill energy demands in a process known as functional hyperemia.

The main players of these dynamic adjustments in the cerebral blood flow are neurons, glia, and vascular cells. All of them come together in a functional morphological entity termed the “neurovascular unit”. This view explains why cerebrovascular diseases linked to neurodegeneration are considered as stand-alone etiopathological states (Przedborski et al., 2003; Brown et al., 2005). Vascular diseases, however, may be the forerunners of neurodegeneration. In support of this, it has been shown that shifts in blood pressure lead neurons to adjust the excitability thresholds according to brain perfusion levels, safeguarding their homeostasis. Hence, vascular diseases may occur first and then later neurodegeneration may follow, if the process of neurovascular coupling proceeds ineffectively (Moore and Cao, 2008; Kim et al., 2016).

Cerebral blood vessels originate from large cerebral arteries arising from the circle of Willis. As they find the way through the brain, large cerebral arteries divide into pial arteries and arterioles, which grow gradually deeper and form the penetrating arteries and arterioles that become progressively smaller until they turn into cerebral capillaries (Wittko-Schneider et al., 2014). The development of nerves and astrocytes originating from central and peripheral sources is closely associated with cerebral arteries, arterioles, and capillaries as demonstrated by the observation that the inhibition of angiogenesis during development is accompanied by severe developmental defects in specific regions of the central nervous system (CNS) (Hallene et al., 2006). This highlights the importance of blood vessels during brain formation.

There are different steps and mediators underlying neurovascular coupling. Briefly, neurons and interneurons are responsible for the initiation and modulation of the vascular response by the activation of NMDA and AMPA receptors, which leads to an increase of intracellular Ca^2+^ and calcium-dependent enzymes that exert effects on the endothelium, astrocytes, and pericytes. Because of their close association with synapses and microvessels, astrocytes are in charge of the neurovascular transmission of signals to the capillaries and arterioles while endothelial cells exert a retrograde propagation of vasomotor responses to regulate cerebral blood flow (Iadecola, 2017). The endothelium also regulates the vascular tone by releasing potent relaxing and contracting factors that readjust the vascular musculature and maintain a healthy homeostasis of the vascular wall.

Nitric oxide (NO), prostacyclins, endothelial-derived hyperpolarizing factor (EDHF), and endothelin have been proposed as the main vasoactive factors related to the endothelial response to neural activity. NO promotes the vasodilation of cerebral blood vessels by stimulating soluble guanylate cyclase in the vascular muscle, leading to an intracellular increase of cGMP and relaxation (Schmidt and Walter, 1994). NO is generated by three different isoforms of the enzyme NO synthase (NOS): neuronal (nNOS), inducible (iNOS), and endothelial NOS (eNOS).

In the CNS, nNOS is mainly located in neurons, astrocytes, and neuronal stem cells and has been implicated in synaptic plasticity and in the central control of blood pressure. Abnormal NO signaling is likely to contribute to a variety of neurodegenerative pathologies such as excitotoxicity following stroke, multiple sclerosis (MS), Alzheimer’s disease (AD), and Parkinson’s disease (PD) (Calabrese et al., 2007).

iNOS expression had initially been shown for macrophages, but it is now clear that it can be induced by cytokines in a variety of cell types in the context of inflammation (Li et al., 1991). During episodes of acute inflammation, the role of NO appears to be protective, while chronic iNOS expression is detrimental. The high levels of NO produced by activated macrophages and other cells may not only be toxic to microbes, parasites, or tumor cells, but may also harm healthy cells (Wong and Billiar, 1995).

Endothelial NOS is mostly expressed in endothelial cells, although the isozyme has been detected in certain neurons of the brain (Caviedes et al., 2017). eNOS-derived NO is a well-known physiological vasodilator and an inhibitor of platelet aggregation and adhesion, but it also inhibits leukocyte adhesion and vascular inflammation, controls vascular smooth muscle proliferation, stimulates angiogenesis, and activates endothelial progenitor cells (Forstermann and Sessa, 2012).

In addition to the production and release of NO, cyclooxygenase produces prostacyclin, which induces the relaxation of cerebral vessels by the activation of adenylate cyclase with the accumulation of cAMP and the activation of potassium channels. Similarly, in large cerebral arteries, the production of EDHF by the cytochrome P-450 monooxygenase metabolism of arachidonic acid causes relaxation by hyperpolarizing the underlying vascular muscle through the activation of potassium channels. The contribution of EDHF increases as the vessel size decreases, with a predominance of EDHF activity in resistance vessels and a compensatory upregulation of hyperpolarization in states characterized by reduced NO availability (Ozkor and Quyyumi, 2011).

Besides vasodilatory factors, under certain conditions, the cerebral endothelium produces vasoconstrictive substances such as endothelins leading to a potent and long-lasting contraction of cerebral vessels dependent on extracellular calcium and the activation of protein kinase C (Faraci and Heistad, 1998).

The alterations of any of the cellular players involved in the neurovascular unit may impair its coupling and functional hyperemia, resulting in an alteration of homeostasis leading to brain dysfunction (Iadecola, 2004).

### Heart and Vascular Diseases and the Origin of Neurodegeneration

According to the World Health Organization, cardiovascular diseases (CVDs) lead to one-third of all annual deaths globally, which is about 17.9 million total deaths (Lim et al., 2012). CVDs are disorders of the heart and blood vessels and include among others coronary heart disease, cerebrovascular disease, and rheumatic heart disease. A large number of studies have reported a strong relationship between indices of vascular pathology (such as carotid intima-media thickness, arterial stiffness, and small-artery remodeling) and cognitive impairment and neurodegeneration (Gorelick et al., 2011).

Because the factors contributing to vascular pathology are often preventable or treatable, targeting them may prove useful for preventing or delaying the onset of neurodegenerative diseases. Epidemiological studies show that treating hypertension and hyperlipidemia reduces the incidence of neurodegenerative diseases (Larsson and Markus, 2018) and slows down the rate of cognitive impairment (Deschaintre et al., 2009). Here, we will focus on the main CVDs known to promote changes in the cerebral microvasculature that lead to neurodegeneration in the long run.

#### Hypertension

Hypertension – or blood pressure at or beyond 130 mmHg/80 mmHg (systolic/diastolic blood pressure) according to the 2017 American College of Cardiology/American Heart Association guidelines (Whelton et al., 2018) – affects one billion people worldwide and leads to nine million deaths every year (Lim et al., 2012). In normotensive individuals, cerebrovascular autoregulation counteracts the cerebrovascular effects of the fluctuations in arterial pressure that occur during normal activities. Cerebral arteries relax when arterial pressure decreases and constrict when arterial pressure rises. The role of this vascular adjustment is the maintenance of a stable cerebral perfusion despite changes in systemic arterial pressure.

Hypertension alters cerebrovascular autoregulation (Sadoshima et al., 1983) by impairing endothelium-dependent relaxation (Faraci and Heistad, 1998). Hypertension promotes vascular hypertrophy, remodeling, and atherosclerosis in large cerebral arteries and lipohyalinosis in penetrating arterioles. These changes are damaging because they reduce the lumen of the vessel and increase vascular resistance (Dickinson, 2001). In humans, longstanding hypertension induces the deposition of collagen and fibronectin as well as elastin fragmentation, leading to an increased stiffness of the wall of large arteries (Henskens et al., 2008). Hypertension also affects neurovascular coupling as shown by a reduction in the normal increase in cerebral blood flow elicited in the posterior parietal and thalamic areas during cognitive tasks in patients with chronic untreated hypertension relative to normotensive individuals (Jennings et al., 2005).

The disruption of the adjustment mechanisms by a sustained increase in the blood pressure has been linked to different neurodegenerative diseases such as stroke (MacMahon et al., 1990). Hypertension is also considered an important modifiable risk factor for late-life cognitive decline and non-amnestic mild cognitive impairment in the mid-aged population (Knopman et al., 2001; Reitz et al., 2007). The Honolulu Asia aging study demonstrated an association between elevated levels of blood pressure in middle age and vascular dementia (Launer et al., 2000).

#### Atherosclerosis

Atherosclerosis, a progressive disease, characterized by the accumulation of lipids and fibrous elements in the large arteries, is a major cause of heart disease and stroke (Banerjee and Chimowitz, 2017).

The damage leading to atherosclerosis is elicited over years and is highly influenced by lifestyle. The pathogenic sequence starts with the recruitment of circulating monocytes into the intima, where they differentiate into macrophages and internalize modified lipoproteins to become foam cells (fat-laden M2 macrophages). Then, chemokines and growth factors induce the proliferation of neighboring smooth muscle cells and the synthesis of extracellular matrix components within the intimal compartment, generating a fibromuscular plaque that undergoes progressive structural remodeling resulting in the formation of a fibrous cap, overlying a lipid-rich, necrotic core accompanied by varying degrees of matrix remodeling and calcification (Lusis, 2000). If a rupture of the plaque occurs, the highly thrombogenic contents of the necrotic core are released to the lumen of the vessel, promoting atherothrombotic occlusion.

Epidemiological and post-mortem studies have shown that the atherosclerosis of the arteries that supply the brain is associated with a two- to threefold increased odds of dementia (Hofman et al., 1997; van Oijen et al., 2007; Wendell et al., 2012; Dearborn et al., 2017). Interestingly, this association is independent of the effects of cerebral infarction (Dolan et al., 2010), which can result from the atherosclerosis-induced occlusion of vessels.

#### Cerebral Small-Vessel Disease

Cerebral small-vessel disease comprises white matter lesions (WMLs), lacunar infarcts, and cerebral microbleeds and is a frequent neuroimaging finding (best seen on magnetic resonance images) in elderly people (de Leeuw et al., 2001).

Factors such as arterial stiffness, also related to aging and hypertension, are considered to expose the small vessels in the brain to highly pulsatile pressure and flow (Poels et al., 2012). This causes thickening of the arterial media of these vessels, which gradually narrows the lumen. In addition, atheroscletoric plaques in large arteries can encroach on the origins of smaller arteries, blocking the blood flow to areas supplied by these arteries. Subcortical white matter is particularly susceptible to such changes, as its blood supply depends on small arteries that get progressively smaller as they descend from the cortex to the deeper parts of the brain. Hypoxia ensues, causing edema, demyelination, and infarction of the white matter.

Cerebral small-vessel disease is associated with an increased risk of cognitive decline, dementia, stroke, balance disturbances, and parkinsonism (Bohnen and Albin, 2011). Individuals with an identical WML pattern present clinically heterogeneous complaints in cognitive and motor performance, ranging from no complaints to dementia and parkinsonism, which means other factors determine the cognitive or motor outcome (de Leeuw et al., 2001). Possible explanations for this heterogeneity are the lack of sensitivity of conventional magnetic resonance imaging to the early loss of microstructural integrity in the normal-appearing white matter (Scheltens et al., 1995), the disagreement on how lesions underlying small-vessel disease are classified (Wardlaw et al., 2013), and the efficiency of compensation mechanisms that prevent further cognitive and motor deterioration (Mondadori et al., 2006).

#### Atrial Fibrillation

Atrial fibrillation (AF) is the most common arrhythmia affecting 1 to 2% of the general population (Andrade et al., 2014). AF is characterized by disturbances that promote ectopic firing and reentrant mechanisms that lead to impaired atrial function [for a review on the pathophysiology of AF, refer to (Andrade et al., 2014)].

AF can lead to reduced cardiac output, intermittent cerebral hypoperfusion, and transient hypertension in the cerebral capillaries (Anselmino et al., 2016). Such changes have been suggested as possible mediators of AF-related brain damage. On the other hand, AF may be a manifestation of a broader systemic illness that involves widespread inflammation and platelet activation, which can also lead to impaired cerebrovascular function (Dietzel et al., 2018).

AF is associated with increased morbidity and mortality and has long been known as one of the main risk factors for embolic stroke (Kamel et al., 2016). It is now also considered a risk factor for dementia. de Bruijn et al. (2015) analyzed the association of prevalent and incident AF with incident dementia in 6514 dementia-free patients, revealing not only a positive association between AF and the development of dementia but also that the association was strongest for younger participants with the longest duration of AF. These findings have been replicated by several studies (Nishtala et al., 2018), some of which have shown that the association between AF and dementia is independent of the occurrence of embolic stroke (Ryden et al., 2019) and the use of anticoagulants (Graves et al., 2017). Future investigation will determine if optimal treatment of AF can prevent or postpone cognitive decline and dementia.

### Vascular Pathology in Neurodegenerative Diseases

The link between vascular risk factors and neurodegenerative disease is strengthened by the evidence showing that vascular and neurodegenerative pathophysiological changes often co-exist in patients with a diverse spectrum of neurodegenerative diseases. Several lines of evidence even suggest that vascular mechanisms are *directly* involved in the pathophysiology of neurodegenerative diseases.

The event that triggers the onset of a stroke, such as the rupture of an atherosclerotic plaque, the embolization of a blood clot, or the damage to a vessel and subsequent bleeding, is often preceded by vascular pathophysiological changes that accumulate over time. One of the earliest changes is endothelial dysfunction induced by oxidative stress (Roquer et al., 2009), which occurs as a result of the aforementioned cerebrovascular risk factors. Immediately after the onset of stroke, a series of overlapping pathophysiological changes occur leading to neuronal death within and beyond the initial area of damage. These include critically low blood flow, the accumulation of free oxygen radicals, the sustained or spreading depolarization of neurons, acidosis, tissue inflammation, damage to the blood–brain barrier (BBB), and regulated cell death (Mergenthaler et al., 2004).

The same vascular mechanisms that lead to acute stroke can also lead to cognitive impairment (Popa-Wagner et al., 2010). In the condition known as vascular dementia (or vascular cognitive impairment), the consequences of these pathophysiological changes are the primary cause of neurodegeneration. They include the development of multiple or strategically located infarcts, damage to the myelin sheath and axons, small cerebral hemorrhages, chronic cerebral hypoperfusion, and brain atrophy (Iadecola, 2013). Each of these represents damaged tissue, and the accumulation of damage eventually manifests as cognitive impairment (Gorelick et al., 2011).

Vascular pathology leading to white matter damage, manifesting as WML, seems to play a particularly important role in the pathogenesis of vascular dementia (Iadecola, 2013). Very often, however, both vascular and neurodegenerative changes contribute to the development of dementia, resulting in a condition known as “mixed” dementia.

The link between vascular pathology and neurodegeneration has also been thoroughly studied in AD. Vascular damage, initiated by vascular and genetic predisposing factors, precedes the accumulation of beta-amyloid (Aβ), which eventually leads to neurodegeneration in AD (Nelson et al., 2016). Neuroimaging studies support this theory, showing that reduced CBF and abnormal neurovascular coupling occurs in elderly people at the risk of AD before they show the hallmarks of the disease such as brain atrophy, cognitive impairment, or accumulation of Aβ (Smith et al., 1999; Bookheimer et al., 2000; Ruitenberg et al., 2005).

Vascular damage may facilitate neurodegeneration by altering a major route through which substances are cleared from the interstitial space of the brain parenchyma. This route is known as the glymphatic system, which facilitates the movement of fluid from perivascular (Virchow-Robin) spaces surrounding pial arterioles to the interstitium into spaces surrounding the brain’s deep veins and then to the cervical lymph nodes (Jessen et al., 2015).

Vascular risk factors cause blockages in and around small arteries, which can manifest as an enlargement of the perivascular spaces (Gouw et al., 2011) that are visible on structural magnetic resonance images (Wardlaw et al., 2013). These enlarged spaces are a sign of impeded interstitial fluid flow and hence impaired clearance of substances from the brain parenchyma, and have been linked to an increased risk of dementia (Buerge et al., 2011; Ramirez et al., 2015) and stroke (Mills et al., 2007; Selvarajah et al., 2009).

Interestingly, the spatial distribution of these enlarged perivascular spaces has been linked to different underlying pathologies. A predominant distribution in the basal ganglia indicates vascular disease caused by hypertension (Selvarajah et al., 2009; Charidimou et al., 2013), while a white matter distribution is related to cerebral amyloid angiopathy (Martinez-Ramirez et al., 2013; Charidimou et al., 2014), caused by the accumulation of Aβ protein in the walls of the cerebral and meningeal vessels.

Cerebral amyloid angiopathy, which leads to intracerebral hemorrhage that aggravates the cognitive impairment (Cordonnier, 2011), is found in more than 80% of AD patients (Jellinger, 2010). Aβ itself is a potent vasoconstrictor (Thomas et al., 1996), worsening the hypoperfusion, which, if severe enough, directly injures neurons. However, even milder hypoperfusion alters protein synthesis and plays a role in neurodegeneration (Iadecola, 2004). The critical role of hypoperfusion is supported by the evidence that the induction of chronic global hypoperfusion in animal models causes memory impairment, the accumulation of Aβ, and synaptic dysfunction (Walsh et al., 2002; Koike et al., 2010; Wang et al., 2010).

Blood–brain barrier dysfunction, which involves damage to pericytes and endothelial tight junction molecules, has been observed in several neurodegenerative diseases, including AD, PD, amyotrophic lateral sclerosis (ALS), and Huntington’s disease (Zlokovic, 2008; Chao et al., 2009; Duran-Vilaregut et al., 2009; Henkel et al., 2009; Kalaria, 2010; Alvarez et al., 2011). Vascular damage worsens the situation, both by leading to further BBB dysfunction and because low blood flow traps potentially neurotoxic proteins such as Aβ that pass through the leaky barrier. These proteins, which include immunoglobulins, plasmin, thrombin, and albumin, further reduce blood flow and cause vasogenic brain edema, oxidative stress, and axonal demyelination (Chen and Strickland, 1997; Mhatre et al., 2004; Farrall and Wardlaw, 2009; Chen et al., 2010). In patients, this BBB dysfunction is detectable using neuroimaging and the analysis of the biomarkers of neurodegenerative diseases in the cerebrospinal fluid (Olsson et al., 2016; Sweeney et al., 2018).

The presence and severity of vascular damage occurring at the cellular and molecular level, affect the clinical course of neurodegenerative diseases. Large neuropathological studies show the post-mortem presence of infarcts, lacunes, leukoencephalopathy, atherosclerosis, or hemorrhages in 80% of AD patients compared to 67% of similarly aged people without cognitive impairment (Toledo et al., 2013).

Interestingly, the effects of vascular pathology and neurodegeneration on cognitive decline seem to be synergistic, particularly in patients with moderate AD pathology (Zekry et al., 2002; Choi et al., 2011; Lee et al., 2014). Studies show that the worse the vascular pathology in patients with AD, the worse the cognitive impairment (Snowdon et al., 1997; Song et al., 2007; White, 2009). AD patients with infarcts or microangiopathic white matter disease also experience faster cognitive decline (Silvestrini et al., 2006; Helzner et al., 2009).

## Cellular Senescence as the Origin of Neurodegeneration

Since a main feature of aging is the accumulation of senescent cells in various tissues (van Deursen, 2014; Calcinotto et al., 2019), cellular senescence has emerged as a key potential contributor to neurodegenerative and cerebrovascular diseases (Baker and Petersen, 2018; Kritsilis et al., 2018).

**FIGURE 1:**
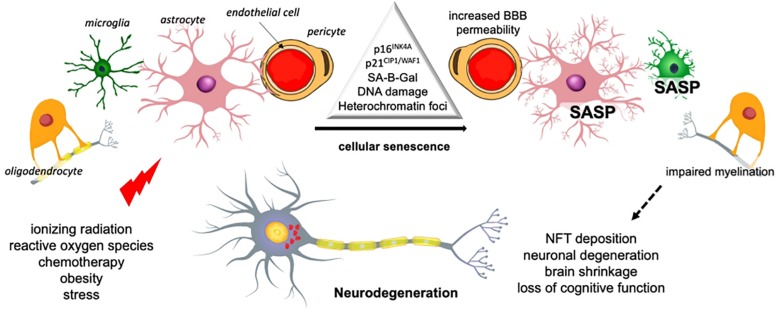
Senescence-mediated neurodegeneration. Cellular senescence can be induced by several stimuli in the different brain cells. Senescence of microglia and astrocytes results in inflammation and the loss of trophic support. Oligodendrocyte senescence reduces myelin, which affects transmission and the BBB may be compromised by endothelial cells senescence. These changes have an impact on the integrity and viability of neurons and ultimately brain function [based on Chinta et al. (2015)]. SASP, senescence-associated secretory phenotype.

Cellular senescence is a phenotype characterized by a durable cell cycle withdrawal (i.e., cells do not respond to mitogens), associated with the expression of tumor suppressors p21^CIP1/WAF1^ (encoded by *Cdkn1a*) and/or p16^INK4A^ (encoded by *Cdkn2a*), as well as by apoptosis resistance (Figure 1). Senescent cells display a flattened and vacuolated morphology with abundant stress granules and accumulate lipofuscin. There is a persistent DNA damage response commonly detected by the presence of γH2AX foci, while the most common feature is an increase in the lysosomal activity of the enzyme senescence-associated beta galactosidase isoform (SA-B-Gal), which has been widely used for their detection. Some senescent cells exhibit a formation of senescence-associated heterochromatin foci (SAHF), which are domains enriched in the transcription-silencing histone H2A variant, macroH2A.

However, the most important feature of senescent cells is the secretion of pro-inflammatory cytokines, growth factors, extracellular enzymes, and metalloproteases, collectively known as senescence-associated secretory phenotype (SASP) (Chow and Herrup, 2015; Ask et al., 2018). In early stages, the secretion of cytokines by senescent cells promotes the migration and infiltration of effector immune cells and the secretion of growth factors and proteases, which facilitate tissue repair and remodeling (Munoz-Espin and Serrano, 2014; Davaapil et al., 2017).

Yet, persisting cytokine signaling, as occurs in aging, contributes among other processes to chronic inflammation (*inflammaging*), a major contributor to age-related dysfunctions. SASP molecules also have an autocrine role, fostering the senescent phenotype, and a paracrine role inducing senescence in surrounding cells (Coppe et al., 2010). Notably, during aging, senescent cells accumulate and persist in different organs, including the brain, and are associated with the onset of several diseases (Munoz-Espin and Serrano, 2014).

To avoid confusion, it is however important to differentiate between aging, often termed “senescence” and cellular senescence. Aging is featured by an accumulation of senescent cells, but not all aged cells have the unique cellular senescence phenotype. Further investigations are needed to understand why senescent cells accumulate with aging, perhaps involving a combination of immune system malfunction failing to eliminate them, with changes in senescent cells themselves so that they become less efficiently recognized.

### Senescence in Neurodegenerative Diseases

More recent *in vitro* and *in vivo* evidence strengthens the hypothesis of cellular senescence as causative of neurodegeneration. Dysfunctional astrocytes with senescent hallmarks have been detected in post-mortem human brain tissue from patients with ALS, PD, and AD (Bhat et al., 2012; Chinta et al., 2015, 2018; Turnquist et al., 2016). Mouse models and *in vitro* studies provide further evidence of senescence in microglia (Bussian et al., 2018), endothelial cells (Broadwell, 1989), neural stem cells and possibly oligodendrocytes (Kritsilis et al., 2018), which may contribute to neurodegenerative disorders.

Moreover, several senescent features have recently been described in studies of both physiological aging and neurodegenerative diseases using different models, including AD, PD, MS, and stroke (Kritsilis et al., 2018; Walton and Andersen, 2019), supporting the idea that cellular senescence may contribute to neurodegeneration and cerebrovascular disease in a far-reaching way (reviewed by Baker and Petersen, 2018; Walton and Andersen, 2019).

In a recent publication, using weighted gene co-expression analysis, Mukherjee et al. (2019) found a microglial signature of 17 genes that are strongly related with both aging and neurodegenerative diseases, not only in human brains but also in neurodegenerative murine models. An early study from Bhat et al. (2012) suggested that p16^INK4A^-positive astrocytes may increase the risk for sporadic AD, since the prefrontal cortex of AD patients harbors a significant increase of senescent astrocytes expressing p16^INK4a^ and matrix metalloproteinase-1 (MMP-1).

Also, the *in vitro* exposure of astrocytes to Aβ_*1–42*_ triggers senescence, leading to the secretion of pro-inflammatory cytokines, including interleukin 6 (Bhat et al., 2012). Chinta and colleagues also demonstrated an increase of senescent astrocytes in brain tissue from PD patients, and found that cultured murine and human astrocytes exposed to paraquat (an herbicide associated with sporadic PD) become senescent. Interestingly, the depletion of senescent cells diminished neurodegeneration (Chinta et al., 2018), suggesting that senescence may be the cause, not the consequence of neurodegeneration.

Cellular senescence has also been considered as a therapeutic target in MS (Oost et al., 2018). Although MS is regarded as a classical autoimmune disease, it has been hypothesized that the driver of the autoreactive immune response in MS is actually a primary neurodegenerative process (Trapp and Nave, 2008). The senescence of microglia, T-cells, astrocytes, endothelial cells, and oligodendrocytes, among other cell types, is implicated in MS pathophysiology (Oost et al., 2018). Even though no study has been conducted for senescent cell detection on post-mortem tissue of MS patients, there is initial evidence in animal models about the role of cellular senescence on MS progression (Oost et al., 2018).

### Senescence and Synaptic Plasticity

Another common hallmark of neurodegenerative pathologies is the alteration in synaptic structure and function during the early stages of the disease, which is related to subcellular reorganization preceding cell loss. The mechanisms underlying these pathologies are not fully understood. In fact, a decline in synaptic plasticity may be linked to the phenomenon of senescence. Unfortunately, there are only a few studies that have investigated the effect of senescence on the synaptic machinery. However, there is growing evidence of the changes in neuroplasticity caused by inflammation, oxidative stress, and protein misfolding, that may be linked to SASP action as well as the influence of senescence in glial cells, which are associated with an impairment in the neuroplasticity machinery (Gan et al., 2018).

The hippocampus has been pointed out as a brain structure with high plasticity and prone to neurodegeneration with age (Kreutzmann et al., 2015). The neurogenic capacity of the hippocampal dentate gyrus is reduced during aging. This reduction correlates with an impairment in memory formation and is accompanied by other changes, such as an increase of pro-inflammatory cytokines as well as a reduction of neurotrophic and vascular factors (Seib and Martin-Villalba, 2015; Fan et al., 2017).

Aging is also associated with changes in the hippocampal perforant pathway and its synapses, with the dentate gyrus being particularly affected (Wenk and Barnes, 2000). A decrease of synapses and changes in the morphology of dendrites and their spines have been linked to atrophy and a dysfunction of the aged hippocampus (Rogers et al., 1984). A synapto-proteome study in old animals revealed a general decrease in the expression of neurotransmission regulation proteins, such as post-synaptic density 95, synaptosomal nerve-associated protein 25, shiga toxin 1, synapsin 1 and 2, synaptophysin, and vesicle-associated membrane protein (Wang et al., 2007; VanGuilder et al., 2010; Orock et al., 2019).

Moreover, a recent breakthrough paper, using a classical model of neurodegeneration, shows the accumulation of senescent astrocytes and microglia, but not neurons, in the brains of mice overexpressing human TauP301S. The genetic clearance of senescent glial cells prevented neurofibrillary tangle (NFT) deposition, the degeneration of hippocampal neurons, brain shrinkage, and the thinning of the dentate gyrus, thus preserving cognitive function. The transgenic mice that did not undergo a senescent transformation of microglia cells maintained their cognitive functions (Bussian et al., 2018).

Interestingly, neurogenesis and learning and memory can be impaired when blood and plasma from aged animals are transfused to young animals, showing that systemic factors can affect brain integrity. This phenomenon may be related to chemokine levels, especially the C–C Motif Chemokine Ligand 2 (CCL2), an important SASP component (Villeda et al., 2011). It is possible that when senescent astrocytes accumulate, they release SASP factors that lead to NFT deposition and other neurodegenerative hallmarks in surrounding neurons, oligodendrocytes, microglia, and endothelial cells (Figure 1). Investigating senescent cell accumulation in the periphery and in the nervous system should help to understand the process of aging of the brain and, more importantly, neurodegeneration in an integrative way.

### Cellular Senescence in Stroke

Cellular senescence accompanying neurological conditions involves many cell types of the CNS (Walton and Andersen, 2019), which may also be of great importance in the case of brain injury following stroke (Buga et al., 2013). Most studies in this field, however, focused on the effects of aging rather than on cellular senescence. The timing of the cellular and genetic response to brain tissue injury is dysregulated in aged animal models due to a premature accumulation of BrdU-positive microglia and astrocytes, activated oligodendrocytes, and degenerated neurons (Popa-Wagner et al., 2007).

There is no consensus whether and to what extent age influences infarct size: several studies reported larger (Popa-Wagner et al., 2011) or smaller ischemic lesions in aged animals when compared to young rodents (Manwani et al., 2013; Ritzel et al., 2018). However, irrespective of the infarct size, aged animals have worse functional when compared to young ones (Popa-Wagner et al., 2011; Ritzel et al., 2018). The slow tissue recovery and rapid development of damage after injuries like stroke in aged animals may be linked to several factors such as a reduced expression of genes linked to neuroprotective pathways, high and accelerated cell death, high oxidative stress by mitochondrial dysfunction, increased permeability of the BBB, high phagocytic activity of brain macrophages, autophagic dysfunction, as well as an imbalanced inflammatory state (Popa-Wagner et al., 2007, 2011; Petcu et al., 2008).

Furthermore, the immune response to ischemic brain injury differs between aged and young mice. In a recent report, older mice displayed an increased number of brain-infiltrating and peripheral neutrophils with decreased phagocytic potential, increased levels of reactive oxygen species and extracellular matrix-degrading enzymes (Ritzel et al., 2018). Rawji and colleagues reported that aging also affects brain-resident immune cells. Aged microglia express excessive amounts of pro-inflammatory cytokines and proliferate at increased rates, but simultaneously display defective phagocytosis and decreased motility. Interestingly, aged peripheral monocytes seem to share the features of impaired phagocytosis and reduced motility, but are less prone to activation and proliferation (Rawji et al., 2016).

Since aging has been linked to the accumulation of senescent cells (van Deursen, 2014; Calcinotto et al., 2019), we speculate that cellular senescence may contribute to the outcome after stroke. However, there is a need for more studies, exploring specifically the impact of cellular senescence rather than the global effects of aging. *In vitro* and *in vivo* experiments show that aging influences the state of endothelial cells, pericytes, and tight junctions, in turn resulting in a compromised function of the BBB (Yamazaki et al., 2016). Acute stroke leads to increased permeability and BBB breakdown, associated with worse outcome in stroke patients (Brouns et al., 2011).

Astrocytes in aged brains display the features of cellular senescence and SASP (Salminen et al., 2011), possibly impacting surrounding cells also in the case of acute brain damage. In the context of senescence hallmarks, human astrocytes are highly sensitive to oxidative stress and trigger a senescence program when faced with multiple types of stressors (Crowe et al., 2016; Liddell, 2017).

Chronic low-grade inflammation contributes to the increased risk of cerebrovascular disease (Lucas et al., 2006) and was shown to be a feature of the aging (Buga et al., 2013). The SASP in many cell types, including peripheral immune cells, astrocytes and microglia may influence a chronic inflammatory state observed in aging (Buga et al., 2013).

Taking into consideration the pathogenesis of cerebrovascular diseases, it is important to mention that cellular senescence may play a role in comorbidities and conditions known as risk factors for stroke (Buga et al., 2013). Cellular senescence impacts the formation and state of atherosclerotic plaques (Ritzel et al., 2018) and endothelial cells showing the hallmarks of senescence triggered by stressors like changes in glucose levels, homocysteine or elevated blood pressure (Tian and Li, 2014) may play a role in age-related vascular diseases. Since cellular stress induces senescence, it remains to be experimentally tested whether stroke itself triggers senescence in brain cells, which may influence the CNS microenvironment, have long-term consequences and an impact on the outcome after cerebral insults.

### Systemic Metabolic Disease, Cellular Senescence, and Neurodegeneration

Risk factors in many neurodegenerative disorders include systemic metabolic diseases (see section “Impact of Peripheral Metabolic Disorders on Neurodegeneration”), which, in fact, favor the accumulation of senescent cells by facilitating the functional changes of cellular distribution and regulation of adipose tissue. As mentioned before, cellular senescence in aging may contribute to a chronic inflammatory state in the brain and peripheral tissues (Buga et al., 2013).

Interestingly, obesity generates an accelerated state of aging, pro-oxidant factors as well as glucotoxicity (Hardie et al., 2006; Eberle and Ament, 2012). It is also associated with an increased senescent cell burden and a wide range of neurodegenerative and neuropsychiatric diseases (Gariepy et al., 2011; Hryhorczuk et al., 2013; Morsi et al., 2018). Ogrodnik and colleagues demonstrated that, in obese mice, glial cells show increased markers of cellular senescence in the periventricular region of the lateral ventricle, a region in close proximity to the neurogenic niche (Ogrodnik et al., 2019).

Furthermore, the affected glial cells in obese mice display excessive fat accumulation, which is in agreement with the role of glial cells on the senescence-mediated induction of neurodegeneration. Since SASP contributes to inflammation, metabolic dysregulation, progeria, pulmonary fibrosis, geriatric syndromes, and the loss of resilience, the clearance of senescent cells may delay or even alleviate multiple age-related diseases (Kirkland and Tchkonia, 2017).

Taking into consideration the increasing body of evidence on the role of cellular senescence in neurodegenerative, cerebrovascular, and metabolic disorders, we propose to investigate senescent cell accumulation in the periphery and in the nervous system as well as the influence of SASP on processes in the CNS, such as neuroplasticity, specific cellular populations, and on the brain–body signaling. This will help to understand the normal process of aging and senescence in an integrative way.

## Body–Brain Trophic Interactions as the Origin of Neurodegeneration

The disruption of the bidirectional interaction between neurons and the cells they innervate has not been considered as a putative etiologic mechanism of neurodegeneration. According to the trophic theory of neural connections, the body is represented several times within the nervous system along the sensory and motor pathways (Purves, 1988). Thus, changes in size and function of the body must be accompanied by changes in their neural representation at a central level, which may include neurite remodeling and functional adaptations.

**FIGURE 2:**
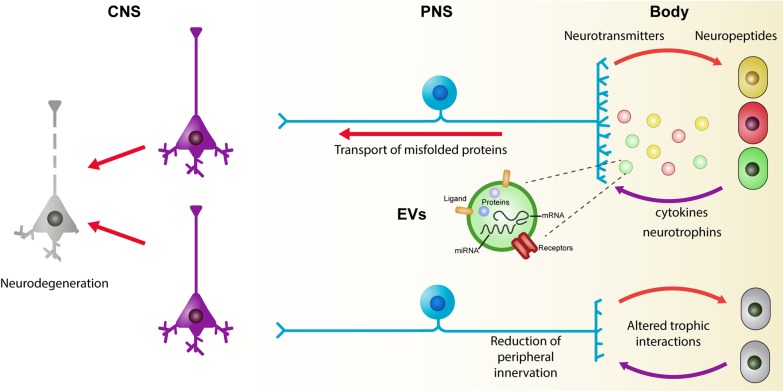
The role of trophic interactions between the brain and the body in the development of neurodegenerative diseases. Under physiologic conditions, there is a continuous exchange of information between neurons and their targets. Neurons control several aspects of the cells they innervate by the release of neuropeptides and neurotransmitters. Reciprocally, peripheral cells modulate neuronal physiology by the release of several molecules including neurotrophins and cytokines. Recently, extracellular vesicles (EVs) have been considered as key players in the development and progression of chronic and degenerative diseases. An alteration of the homeostatic state of peripheral cells is reflected on the molecular cargo of EVs which, in turn, can affect the functioning of the neurons. Common to several chronic and neurodegenerative diseases, there is a reduction of peripheral innervation which leads to a disturbance of the trophic relation between the body and the central nervous system. A continuous alteration of these interactions during maturity of individuals may underlie some of the early events that reflect on neuronal death during aging. CNS, central nervous system; EVs, extracellular vesicles; PNS, peripheral nervous system.

Furthermore, novel requirements of the body affect the peripheral and central connections of peripheral neurons. In early phases of metabolic disorders, adipocytes and immune cells are related to peripheral nerve remodeling of both sympathetic and sensory fibers (Pellegrinelli et al., 2018; Yamazaki et al., 2018). In addition, the alteration of the enteric nervous system (ENS) may be a conduit to induce changes in the CNS. Future studies oriented to understand the interactions between neurons and the cells they innervate would help to discover biomarkers for early diagnosis and to monitor disease progression. Here, we present a brief summary of the experimental evidence related to the early events of neurodegeneration in the body and discuss possible mechanisms of intercellular communication that may be operating (Figure 2).

### Pathological Events in the Peripheral Nervous System

Various neurodegenerative diseases share common molecular and cellular mechanisms including the misfolding of proteins that undergo conformational changes leading to tissue deposition and the formation of inclusion bodies. Increasing evidence suggests that these neurodegenerative proteinopathies may originate in the peripheral nervous system (Wakabayashi et al., 2010) or even across the body.

In PD and dementia with Lewy bodies (DLBs), the histopathological hallmark is the occurrence of neuronal α-synuclein aggregates, also known as Lewy bodies. Lewy bodies are typically found in the substantia nigra in PD. However, LBs can also be present in peripheral organs, neural or not, including the sympathetic ganglia, ENS, cardiac and pelvic plexuses, submandibular gland, adrenal medulla, and the skin (Beach et al., 2010; Wakabayashi et al., 2010). Moreover, in the early pathological stages of PD, α-synuclein pathology is present first in the dorsal motor nucleus of the glossopharyngeal and vagal nerves as well as the anterior olfactory nucleus (Braak et al., 2003a).

In AD, NFTs are observed in the spinal and sympathetic ganglia. However, it seems that they develop independently of the cerebral AD pathology (Shankle et al., 1993). In ALS, TDP-43 is a major component of the ubiquitinated inclusions and also occurs in the neurons of the spinal ganglia (Nishihira et al., 2008).

Sensory abnormalities have been described in neurodegenerative diseases including PD and AD. In this regard, the loss of olfaction represents an important early clinical symptom frequently preceding motor and cognitive impairments typically observed in these neurodegenerative diseases (Driver-Dunckley et al., 2014; Doty, 2017; Marin et al., 2018; Rey et al., 2018). The olfactory system may be particularly affected in neurodegenerative diseases as seen by the accumulation of pathological protein aggregates in olfactory structures including the anterior olfactory nucleus, the olfactory bulb, and the olfactory epithelium (OE) (Doty, 2017; Marin et al., 2018; Rey et al., 2018).

Given that the OE is directly accessible to external factors, it is vulnerable to environmental insults. Thus, external agents (toxins, bacteria, viruses, air pollutants) may trigger neurodegeneration through the OE (Rey et al., 2018). Once these agents enter the brain via the OE, they may cause protein misfolding and the spreading of protein aggregates in a prion-like manner throughout the olfactory pathway to other anatomically connected brain regions (Doty, 2017).

Moreover, xenobiotics and particles in contact with the OE can also induce the production of reactive oxygen species triggering a local inflammatory reaction in a similar way to that induced by misfolded proteins (Lema Tome et al., 2013). Alternatively, the enhanced expression of misfolding-prone proteins in olfactory structures may make those regions prone to develop certain diseases (Duda et al., 1999; Taguchi et al., 2016; Kim et al., 2018).

Pain abnormalities are part of the clinical features in patients with PD and the impaired nociceptive processing depends on nociceptor degeneration (Nolano et al., 2008; Reichling and Levine, 2011; Conte et al., 2013). Skin biopsies of PD patients reveal a decrease in cutaneous autonomic innervation, free epidermal nerve endings, and encapsulated sensory endings (Dabby et al., 2006; Nolano et al., 2008). The severity of PD correlates with the loss of Meissner corpuscles and epidermal nerve fibers, accompanied by a reduction in cold and pain perception (Nolano et al., 2008).

The accumulation of misfolded proteins such as α-synuclein and parkin due to a dysfunction of the ubiquitin-proteasome system may partially explain the painful nociceptor dysfunction (Saha et al., 2000). In AD, pain perception seems not to be reduced. However, more studies are necessary to document whether sensory innervation is modified in AD and other neurodegenerative diseases (Cole et al., 2006). Of note, it is important to mention that different mechanisms involving both peripheral and central pain pathways may play a role in sensory dysfunction.

Autonomic dysfunction is present in several neurodegenerative diseases including PD, multiple system atrophy, AD, and other types of dementia such as DLB, PD with dementia, and frontotemporal lobar degeneration (Idiaquez and Roman, 2011; Coon et al., 2018). Orthostatic hypotension has been widely reported in patients with PD and DLBs as seen by the decreased cardiac uptake of the noradrenaline analog meta-iodobenzylguanidine (Orimo et al., 2005).

The presence of orthostatic hypertension may be due to the depletion of cardiac vagal sympathetic nerves as well as the α-synuclein pathology in sympathetic ganglia and adrenal gland that have been reported in Lewy body diseases, which includes PD, PD with dementia, and DLB (Wakabayashi et al., 2010). Interestingly, sympathetic denervation precedes neuronal loss in the sympathetic ganglia (Orimo et al., 2005). Autonomic dysfunction in AD may also be affected by neuropathological changes in central autonomic networks (Collins et al., 2012). Future studies should focus on the temporality of autonomic alterations in neurodegenerative diseases.

Gastrointestinal dysfunction is closely linked to the dysfunction of the ENS. Enteric dysfunction has been reported in PD, AD, ALS, and frontotemporal dementia (Rao and Gershon, 2016). Biopsies of PD patients have demonstrated the presence of Lewy-type pathology in enteric neurons suggesting a role of the ENS in the onset of the disease (Kupsky et al., 1987; Wakabayashi et al., 1988, 1990). The unmyelinated axons of enteric neurons may increase their susceptibility to PD and due to their multiple synaptic terminals may promote the propagation of α-synuclein aggregates via connected ganglia (Chalazonitis and Rao, 2018).

Likewise, in AD patients, Aβ immunoreactivity is found in the submucosa of the intestine (Joachim et al., 1989). In addition, amyloid precursor protein expression is present in the enteric neurons of AD patients (Arai et al., 1991). However, in contrast to PD, data from human samples in AD are still sparse. Other autonomic dysfunctions reported include urinary symptoms, sexual and thermoregulatory, dysfunction, and sleep disturbances (Postuma et al., 2015; Racosta et al., 2015; Pfeiffer, 2016). A better characterization of peripheral damage is required in those cases where there is evidence of alteration in the peripheral innervation.

### Impact of Peripheral Metabolic Disorders on Neurodegeneration

An increasing body of evidence suggests that metabolic disturbances may contribute to neurodegenerative processes in the CNS. We discuss here briefly the impact of diabetes mellitus (DM) and obesity on neurodegeneration.

Diabetes mellitus is one of the most common metabolic diseases and a major disorder of insulin regulation with increasing incidence (Duarte et al., 2012). Especially type 2 diabetes (T2D) is a highly complex, multifactorial metabolic disease, characterized by a progressive pancreatic β-cell failure (relative insulin deficiency), decreased insulin action and peripheral insulin resistance (Campbell, 2011; Carvalho et al., 2012).

T2D develops under a cluster of risk factors that includes high blood glucose, obesity, increased blood triacylglycerols and insulin resistance, which individually or collectively, also increase the risk for neurodegeneration/neuronal death, functional and structural brain changes, culminating in cognitive dysfunction that underlies dementia-type disorders (e.g., AD), which may arise from a complex interplay between T2D and brain aging. Additionally, decreased brain insulin levels/signaling and glucose metabolism in both pathologies further suggests that an effective treatment strategy for one disorder maybe also beneficial in the other (Duarte et al., 2013).

Obesity is clinically identified based on measurements of body mass index, but can be generally defined as the condition in which excess body fat has accumulated to an extent that can negatively affect health. This definition is based on the dramatically enhanced risk for a myriad of disorders, including T2D, CVD, gastrointestinal and respiratory disorders, and several types of cancer (for review, see Haslam and James, 2005). Furthermore, abdominal obesity is a component of the metabolic syndrome, which additionally combines insulin resistance or glucose intolerance, atherogenic dyslipidemia, elevated blood pressure, and increased expression of prothrombotic and proinflammatory markers (for review, see Olufadi and Byrne, 2008) and is an important risk factor for T2D, CVD, and stroke.

While the consequences of obesity on metabolic and cardiovascular pathophysiology are well-studied, epidemiological and experimental data are beginning to reveal that the CNS may also be detrimentally affected by obesity and obesity-induced metabolic dysfunction. In particular, data show that obesity is associated with cognitive decline and enhanced vulnerability to brain injury, while experimental studies in animal models confirm a profile of increased susceptibility to brain damage and decreased cognitive function (Bruce-Keller et al., 2009).

Recent data suggest that both AD and PD can manifest systemic alterations in energy metabolism (e.g., increased insulin resistance and dysregulation of glucose metabolism). Moreover emerging evidence that dietary restriction can forestall the development of AD and PD is consistent with a major “metabolic” component to these diseases, and provides optimism that these devastating brain disorders of aging may be largely preventable (Blum-Degen et al., 1995; Mattson et al., 1999).

Advanced glycation end products generated by chronic hyperglycemia and their receptor RAGE provide critical links between diabetes and AD (Vicente Miranda et al., 2016; Fleitas et al., 2018). Obesity, diabetes, and metabolic syndrome increase the risk of cognitive decline and dementia, including not only vascular dementia, but also AD and PD (Roriz-Filho et al., 2009).

However, due to increased incidence, especially in the aging population, the impact of metabolic disease on neurodegenerative processes will increase tremendously in the upcoming decades. We believe therefore that potential therapeutic approaches aiming at preventing/treating neurodegenerative diseases should include metabolic disorders.

### Altered Body–Brain Trophic Communication

Accumulating evidence suggests that patients with neurodegenerative diseases commonly develop sensory and autonomic dysfunctions during maturity. This raises the possibility that the peripheral nervous system may be the gateway to alter brain physiology. The trophic theory of neural connections states that there is a mutual dependence between neurons and their targets (Purves, 1988; Qureshi and Mehler, 2013; Holm et al., 2018). Olfactory or gastrointestinal alterations may reflect a distortion of the communication between a variety of cell types and peripheral neurons. The repercussion of these altered interactions on the development of neurodegenerative diseases are still underexplored. Moreover, the mechanisms of cellular communication between peripheral cells and the nervous system are not fully characterized.

Recently, a novel system of intercellular communication has been proposed to modulate changes in neuronal physiology and to participate in the propagation of misfolded proteins (Fevrier et al., 2004; Asai et al., 2015; Grey et al., 2015; Polanco et al., 2016). This system is mediated by membranous organelles denominated as EVs, which can travel from the periphery to the brain and vice versa (Li et al., 2018).

Apparently, all the cell types of the body can release EVs to transmit information in the form of proteins, RNAs, and lipids. EVs are classified according to their subcellular origin in exosomes when originating from multivesicular bodies and in ectosomes (or microvesicles) when formed from the plasma membrane (Thery et al., 2018). Despite the technical challenges to study the functional role of different EVs subpopulations, their participation in both the maintenance of tissue homeostasis and the progression of chronic degenerative diseases is becoming clearer.

Extracellular vesicles are involved in several processes of brain development and normal functioning, including synapse formation, synaptic plasticity, and communication between glia and neurons. The signaling proteins of hydrophobic nature, such as the Wnt family members and other regulatory proteins, are loaded into EVs (Korkut et al., 2009; Coulter et al., 2018; Lee et al., 2018). During mammalian brain development, the vesicular secretion of the growth factor sonic hedgehog promotes the proliferation of neural progenitors.

Interestingly, the loss of function of the complex charged multivesicular body proteins 1A reduces the number of intraluminal vesicles and the secretion of a specific subtype of EVs whose functions can be directly related to sonic hedgehog signaling (Coulter et al., 2018). At the *Drosophila* neuromuscular junction, EVs containing the Wnt-binding protein Evennes Interrupt and the Wnt 1 homolog Wingless promote synaptic growth when released from the presynaptic terminal (Korkut et al., 2009; Koles et al., 2012).

There is a constant reciprocal exchange of information through EVs between the pre- and post-synapse to modulate fine aspects of synaptic maturation and remodeling. In rat hippocampal neurons, excitatory synapses can be eliminated by a mechanism dependent on the delivery of Proline-Rich 7 by exosomes to induce the degradation of the post-synaptic density protein-95 (Lee et al., 2018).

Extracellular vesicles contents also influence long-term memory and synaptic plasticity. In neuronal cultures from mouse hippocampus and in neuromuscular junction preparations from *Drosophila*, oligomers of the activity-regulated cytoskeleton-associated protein and its own mRNA are transported through exosomes to the post-synapse (Ashley et al., 2018; Pastuzyn et al., 2018). The translation of the exosomal activity-regulated cytoskeleton-associated protein mRNA increases by the activation of the group 1 metabotropic glutamate receptors (Pastuzyn et al., 2018). The notion that neurons constantly use EV-mediated communication is reinforced by the fact that the release of EVs is activity-dependent and that the delivery of the cargo from presynaptic cells enables retrograde signaling from the post-synaptic cells (Lachenal et al., 2011; Korkut et al., 2013; Lee et al., 2018).

The general hypothesis on the role of EVs related to the development of neurodegenerative diseases relies on their potential to transport protein aggregates, which has been reviewed elsewhere (Howitt and Hill, 2016). Here, we would like to center the discussion on the possibility that a bidirectional exchange of information through EVs between peripheral cells and neurons may have a long-term impact on the brain physiology.

Emerging evidence suggests that both central and peripheral neurons can uptake EVs from a variety of cell types. Thus, under certain circumstances, EVs derived from peripheral cells contain proteins or RNAs that can be deleterious for neuronal functioning. In agreement with this notion, exosomes derived from hyperglycemic Schwann cells contain microRNAs that suppress axonal growth *in vitro* (Jia et al., 2018). Moreover, hyperglycemic Schwann cell-derived exosomes contribute to the development of diabetic peripheral neuropathy when injected into a diabetic mouse (Jia et al., 2018).

Similarly, the CNS can also be affected by the information transported by systemic exosomes. An increase of inflammatory cytokines in the brain and microglial and astrocytic activation is observed after the intravenous injection of serum-derived exosomes from lipopolysaccharide (LPS)-challenged mice (Balusu et al., 2016; Li et al., 2018). Dorsal root ganglion neurons modulate immune cells in the periphery by the release of neuropeptides.

Recent findings also indicate that EV-mediated communication can be an additional mechanism operating during trauma. After nerve injury, dorsal root ganglia neuron-derived exosomes loaded with microRNA-21 increase the infiltration of inflammatory macrophages, which contributes to neuronal sensitization (Simeoli et al., 2017). Further work is required to obtain insights whether EVs from peripheral cells modulate cellular processes in the brain under physiological conditions and which factors can alter the messages coming from the periphery (Ridder et al., 2014).

Despite the few studies focusing on the influence of peripherally derived EVs on peripheral neurons, it is likely that peripheral terminals establish a bidirectional interaction with the cellular elements surrounding them. Both sensory and autonomic axon terminals usually end as free nerve endings in the extracellular matrix intercalated between different cell types. Peripheral neurons are responsive to EVs derived from mesenchymal stem cells and Schwann cells (Lopez-Verrilli et al., 2013, 2016; Ching et al., 2018; Sun et al., 2018; Shiue et al., 2019). The general observation of these studies, performed in conditions of neuronal damage, is that EVs reduce the inflammatory state and have a beneficial role in axonal growth both *in vivo* and *in vitro*.

Taken together, these findings indicate that neurons are susceptible to modify their functioning or gene expression profile as a result of incorporating EVs from neighboring cells. A challenge for future studies is to determine if EVs from any cell source can modify neuronal functioning and how these events are associated with physiological alterations that precede neurodegeneration in the brain.

## Gut Dysbiosis as the Origin of Neurodegeneration

Microbiota is defined as a community of all microorganisms living in our body. Microbiota therefore includes all taxonomic domains such as fungi, protozoa, viruses, and bacteria. Bacterial microbiota living in the gut (further referred as GM) represent about 90% of all microbiota in the body (Thursby and Juge, 2017), with an estimated ratio of human:bacterial cells close to 1:1 (Sender et al., 2016). This large number of bacteria offers different benefits to the host, confers protection against pathogens (Baumler and Sperandio, 2016), regulates host immunity (Gensollen et al., 2016), and products originating from bacterial fermentation can be used as a source of energy for colonocytes (den Besten et al., 2013).

A delicate balance exists between the GM composition and its interaction with the host, while an imbalance in this composition is commonly known as dysbiosis. Gut dysbiosis has been implicated in the development of chronic diseases, such as irritable bowel syndrome (Raskov et al., 2016), coeliac disease (Verdu et al., 2015) as well as metabolic disorders, including obesity, metabolic syndrome, and T2D (Holmes et al., 2011; Carding et al., 2015; Zhang et al., 2015; Baothman et al., 2016; Org et al., 2017).

Notably, those metabolic diseases, when developed during adulthood, are considered important risk factors for AD (Alzheimer’s Association, 2016; Livingston et al., 2017). Furthermore, metabolic alterations are known to induce peripheral low-grade chronic inflammation, associated with higher incidence of neurodegenerative diseases. In AD, systemic inflammation during prodromal stages shortens disease onset (Tao et al., 2018), whereas dysregulation of the gastrointestinal function and colonic inflammation diminished immune competence and preceded motor symptoms in PD patients (Dobbs et al., 1999; Braak et al., 2003b; Lebouvier et al., 2009; Villaran et al., 2010; Forsyth et al., 2011).

During aging, there is an increased permeability of the gut epithelium (Tran and Greenwood-Van Meerveld, 2013). This condition allows bacteria and bacterial products to translocate through the disrupted gut barrier reaching peripheral organs (Zevin et al., 2016). Newest reports suggest that translocated bacteria may even reach the brain (Roberts et al., 2018), where an exacerbated immune reaction can lead to neurodegeneration. These observations remain however to be investigated in detail.

### Gut Microbiota Alterations in Neurodegenerative Diseases

Recent reports concomitantly demonstrate a gut dysbiosis in PD (Minato et al., 2017; Wu et al., 2017), ALS, and AD patients (Cattaneo et al., 2017; Vogt et al., 2017).

Understanding how GM may be implicated in brain pathologies requires a clear comparison among the data reported from different research groups. The GM is mainly dominated by four phyla: Firmicutes, Bacteroidetes, Actinobacteria, and Proteobacteria (Szablewski, 2018). The first studies described GM alterations only at the phylum level. However, recent advances in sequencing techniques allow distinction at the species level, which is advantageous since the different species of bacteria belonging to the same genus affect host physiology in a different manner. We compared previously published GM data obtained from AD patients and AD mice models. We noted a strong variation between studies: at phylum level, most transgenic models coincide with an increased abundance of Firmicutes but a decreased abundance of Bacteriodetes (Table 1). Whereas at the family and upper taxonomical levels (i.e., genus/species), there is no more consistency (Table 2).

**TABLE 1:** Alterations in gut microbiota at PHYLUM level in AD patients and AD transgenic mice.

**Model**	**Bacterial phylum INCREASED**	**Bacterial phylum DECREASED**	**References**
AD patients	Actinobacteria	Bacteroidetes	Zhuang et al., 2018
AD patients	Bacteroidetes	Firmicutes	Vogt et al., 2017
3×TG-AD mice 8 months old	Firmicutes	–	Sanguinetti et al., 2018
3×Tg-AD mice 24 months old	–	Tenericutes Cyanobacteria	Bonfili et al., 2017
APP/PS1 mice 8 months old	Firmicutes	Bacteroidetes Proteobacteria	Park et al., 2017
APP/PS1 mice 1 month old	Firmicutes	Bacteroidetes	Harach et al., 2017
APP/PS1 mice 8 months old	Bacteroidetes Tenericutes	Firmicutes Verrucomicrobia Proteobacteria Actinobacteria	Harach et al., 2017
5×FAD mice 9 weeks old	Firmicutes	Bacteroidetes	Brandscheid et al., 2017

**TABLE 2:** Alterations in gut microbiota in AD patients and AD mice at FAMILY, GENUS, and SPECIES levels.

**Model**	**Bacterial family (f.)/genus (g.)/ species (g. s.) INCREASED**	**Bacterial family (f.)/genus (g.)/ species (g. s.) DECREASED**	**References**
AD patients	*g. Escherichia*/*Shigella*	*g.s. Eubacterium rectale*	Cattaneo et al., 2017
AD patients	*f. Bacteroidaceae* *f. Rikenellaceae* *f. Gemellaceae* *g. Bilophila* *g. Phascolarctobacterium* *g. Gemella* *g. Bacteroides* *g. Alistipes*	*f. Ruminococcaceae* *f. Turicibacteraceae* *f. Peptostreptococcaceae* *f. Clostridiaceae* *f. Mogibacteriaceae* *f. Bifidobacteriaceae* *g. SMB53* *g. Dialister* *g. Clostridium* *g. Turicibacter* *g. cc115*	Vogt et al., 2017
AD patients	*f. Ruminococcaceae* *f. Enterococcaceae* *f. Lactobacillaceae*	*f. Lanchnospiraceae* *f. Bacteroidaceae* *f. Veillonellaceae*	Zhuang et al., 2018
3XTG-AD mice 24 months old	*g. Prevotellaceae* *g. Enterococcus* *g. Streptococcus* *g. Turicibacter* *g. Ruminococcus* *g. Desulfovibrionaceae* *g. Flexispira*	*g. Turicibacter* *g. Prevotella* *g. Ruminococcus* *g. Flexispira*	Bonfili et al., 2017
APP/PS1 mice 8 months old	*f. Rikenellaceae* *f. S24-7*	*g. Allobaculum* *g. Akkermansia*	Harach et al., 2017
APP/PS1 mice 8 months old	*f. Aerococcaceae* *f.Leuconostocaceae* *f. Lactobacillaceae* *f. Pseudomonadaceae* *f. Caulobacteraceae* *f. Cytophagaceae* *f. Sphingobacteriaceae* *f. Corynebacteriaceae*	*f. Sphingomonadaceae* *f. Comamonadaceae* *f. Rhodocyclaceae* *f. Flavobacteriaceae*	Park et al., 2017
3XTG-AD mice 8 months old	*f. Enterococcaceae* *f. Turicibacteraceae*	*f. S24-7* *f. Bifidobacteriaceae*	Sanguinetti et al., 2018
3XTG-AD mice 9 months old	*g.s. Prevotella copri* *g.s. Lactobacillus ruminis g.s. Streptococcus anginosus* *g.s. Actinobacillus parahaemolyticus* *g.s. Haemophilus parainfluenzae*	*g.s. Bacteroides fragilis* *g.s. Faecalibacterium prausnitzii* *g.s. Akkermansia muciniphila*	Syeda et al., 2018

Importantly, variations in the experimental design or the type of samples can be observed. Moreover, differences in the animal model, chronological stage of the pathology (age), handling and diet significantly impact GM (Mueller et al., 2006; Hufeldt et al., 2010; Sanchez-Tapia et al., 2017). Thus, experimental variations among published studies may explain discrepancies in the GM composition in AD.

Despite this strong variation, we can observe a general increased abundance of pro-inflammatory bacteria in AD mice and AD patients (Kountouras et al., 2009; Zhan et al., 2016; Cattaneo et al., 2017; Shen et al., 2017; Vogt et al., 2017; Zhang et al., 2017; Syeda et al., 2018) compared to controls. Furthermore, in AD transgenic mice, longitudinal studies clearly indicate an age-associated gut dysbiosis, culminating in a strong pro-inflammatory environment along the development of brain pathological hallmarks (Shen et al., 2017; Zhang et al., 2017; Syeda et al., 2018).

### How Can the GM Affect Brain Function or Even Cause Neurodegeneration?

A bidirectional communication between the gastrointestinal system and the brain has been described and has led to the concept of gut-brain axis (Collins and Bercik, 2013). Recent studies illustrated the role of GM in brain Aβ aggregation, as GM absence in germ-free condition (Harach et al., 2017) or GM partial depletion by antibiotic treatment (Minter et al., 2016) reduced the amyloid burden and microglia activation in AD transgenic mice. However, it is still unclear which factors may be associated with the above described gut-brain axis in brain pathologies.

Several groups have implicated the neurotoxin LPS present in the outer membrane of gram-negative bacteria. LPS plays key roles in the host-pathogen interactions of the innate immune system (Hill and Lukiw, 2015; Maldonado et al., 2016). Alterations in the GM composition/abundance are associated with enhanced plasma levels of LPS (Avila-Nava et al., 2017; Sanchez-Tapia et al., 2017), which may result in an exacerbated metabolic endotoxemia (Cani et al., 2012), a condition characterized by inflammation and the increased release of pro-inflammatory cytokines.

Systemic infections have been related to a higher possibility to develop AD (Holmes et al., 2009) as well as early motor dysfunction in PD patients (Dobbs et al., 1999). Therefore, long-term gut microbiota alterations and gut dysbiosis resulting in a pro-inflammatory environment and inflammation may be linked to brain dysfunction in aging.

As a proof of concept, LPS is detected in the parenchyma and blood vessels of both, non-demented aged and AD brain samples, but at a higher level in diseased tissues (Zhao et al., 2015, 2017; Zhan et al., 2016). LPS also increases in the plasma of AD transgenic mice compared to wild type controls (Syeda et al., 2018). In PD patients, higher plasma LPS correlates with α-synuclein aggregation (Forsyth et al., 2011). Furthermore, LPS is a well-known neuroinflammatory agent driving the generation of Aβ_*1–42*_ (Lee et al., 2008; Asti and Gioglio, 2014; Hill and Lukiw, 2015; Zhao et al., 2015). LPS administration to mice results in memory impairment, Aβ aggregation, and astrocyte activation (Lee et al., 2008). Therefore, gut dysbiosis may enhance LPS to the blood circulation and even to the brain, producing glia activation and protein aggregation, both important features of neurodegenerative diseases.

Another bacterial product related to the development of neurodegenerative diseases are short-chain fatty acids (SCFAs). SCFAs are produced by the GM after the degradation of non-digestible polysaccharides, with butyrate, acetate, and propionate being the more abundant fermentation products (Wong et al., 2006). About 95% of the SCFAs produced in the gut are absorbed within the colon (Hoyles et al., 2018), while butyrate is used by the colonocytes as an energy source. In order to maintain an optimal equilibrium between intestinal SCFAs and the concentration in the body, the gut intestinal barrier increases its mucus production (Rios-Covian et al., 2016), while the liver clears the majority of propionate and butyrate from the portal circulation (Bloemen et al., 2010). However, SCFAs can be transported through the bloodstream to reach the brain.

Some alterations in fecal SCFAs concentration have been described in AD transgenic mice compared to controls (Bonfili et al., 2017; Zhang et al., 2017). Butyrate, a well-known neuroprotective agent (Minamiyama et al., 2004; Govindarajan et al., 2011; Liu et al., 2015), is reduced in the fecal samples of AD mice (Zhang et al., 2017). Other reports show increased propionate levels in brain tissue of AD transgenic mice compared to wild type controls, an event associated with GM dysbiosis (Syeda et al., 2018). Although, propionate has some beneficial effects [e.g., on the BBB (Hoyles et al., 2018)], excessive propionate alters dopamine, serotonin, and glutamate systems in a manner similar to that observed in autism spectrum disorders (El-Ansary et al., 2012; Li et al., 2017).

Fecal samples of children with autism spectrum disorder present an increased level of propionate (Wang et al., 2012). In addition, genetic diseases that affect the function of the enzyme propionyl-CoA carboxylase (such as propionic acidemia) are characterized by high levels of propionate and high incidence of dementia (Grunert et al., 2013), while in AD patients, increased levels of acetate and propionate have been found in saliva samples compared to healthy controls (Figueira et al., 2016; Yilmaz et al., 2017). Although, the effects of butyrate and propionate are still controversially discussed, several data support a role of the imbalance between butyrate and propionate in the pathogenesis of AD (Syeda et al., 2018).

Another important pathway connecting the brain and the GM is the involvement of the immune system (Fung et al., 2017) studied, e.g., in stroke. According to recent reports, in the experimental rodent models, stroke leads to dysbiosis (Benakis et al., 2016; Houlden et al., 2016; Singh et al., 2016; Stanley et al., 2018) and dysbiosis may have an effect on the course of the disease (Benakis et al., 2016; Singh et al., 2016; Stanley et al., 2016). This may be linked to GM-related fine-tuning of the balance between pro-inflammatory/anti-inflammatory immune responses primarily in the gut, followed by an infiltration of immune cells to the CNS after injury. Interestingly, pro-inflammatory T-helper cell 17 and γδ T cells found in the ischemic CNS originate from the intestine and the infiltration of these subpopulations has been linked with unfavorable outcome (Benakis et al., 2016; Singh et al., 2016).

However, the optimal (in terms of prognosis after stroke) composition of the GM has not been identified yet and is possibly difficult to pinpoint. Some findings indicate that the complete absence (in germ-free mice) or deep antibiotic depletion of GM can be detrimental, leading to increased infarct volumes (Singh et al., 2018) or increased mortality not related to lesion size (Winek et al., 2016). Alterations in GM have also been identified in stroke patients (Yin et al., 2015; Stanley et al., 2016). Due to its metabolic capacities, GM can also contribute to the pathogenesis of the diseases considered as the risk factors of stroke, like obesity (for review Torres-Fuentes et al., 2017) and atherosclerosis (Karlsson et al., 2012; Tang et al., 2013) (for review, see Li and Tang, 2017).

We can conclude that a predominantly pro-inflammatory GM and the release of neurotoxic substances may negatively affect brain functions, causing systemic and central inflammation over decades. This is in support of the recent hypothesis of an infectious etiology for neurodegenerative diseases, such as AD (Bhattacharjee and Lukiw, 2013; Little et al., 2014; Maheshwari and Eslick, 2015; Itzhaki et al., 2016). The substances released from the GM (i.e., LPS and SCFAs) may be implicated in the pathogenesis of several neurodegenerative diseases; however, more data are still needed to clearly understand this gut-brain-immune interaction.

## From Consequences to Causes: Academic and Preclinical Considerations

As we have described above, the neurocentric view entertained as the predominant focus of our effort in treating neurodegeneration is likely insufficient to ensure successful therapeutic intervention.

### The Lack of Adequate Experimental Models to Study the Etiology

Investigating the pathophysiological processes behind neurodegeneration in detail requires the use of animal models. A plethora of animal models of neurodegeneration exists, and the nuances of these models are beyond the scope of this manuscript (for recent reviews, see Gitler et al., 2017; Dawson et al., 2018; Ransohoff, 2018).

However, two general problems with current models seem to be particularly widespread and contribute to our limited success in extrapolating findings from preclinical research to patients. Firstly, they often fail to fully integrate our understanding of the etiology of the diseases they are meant to represent, often being restricted to inducing the disease in a single way, despite multiple inducing factors being responsible for the human disease. Secondly, animals used as the model organism of specific diseases often do not exhibit similar comorbidities and demographic characteristics (such as age or gender) seen in human patients. Examples of such problems are the rodent models of stroke, particularly ones that involve surgical ligation or occlusion of blood vessels in otherwise healthy, young animals (van der Worp et al., 2010; Mergenthaler and Meisel, 2012).

Another important issue is the way these models are characterized. For animal models to be used to study neurodegeneration, two essential steps must be taken. First, the validity of the model for the question at hand should be thoroughly and systematically investigated. This is preferably done in a multi-centric and multi-disciplinary way to ensure the generalizability and robustness of the results. Second, the external (face, construct, and predictive) validity of the model (Willner, 1984) should be empirically and thoroughly tested, as well as the inter-rater agreement on, and replicability of, the model’s characteristics (McKinney and Bunney, 1969). Such studies aimed at characterizing the model should be very clearly and unequivocally separated from experiments using the model to test hypotheses, such as the effect of an intervention on the course of the model’s progression.

For these strategies to be successful, research into and using these models must be conducted in the setting of appropriate, rigorous, and transparent experimental design and reporting (Kilkenny et al., 2010; Holman et al., 2016; Percie du Sert et al., 2017). Beyond that, we should make an active effort to think of the drawbacks of the models and methods we have used and how this may restrict or bias the knowledge we gain from such studies (Garner et al., 2017). Finally, we need a framework for integrating the knowledge gained from individual studies using different models and methods to address similar research questions. Systematic reviews and quantitative meta-analyses, widely used for knowledge synthesis in clinical research, may be helpful tools to help us achieve this.

### Studying Human Disease in Human Systems

Although animal models are a powerful tool toward understanding the etiology of neurodegenerative diseases, they still present considerable limitations (Jucker, 2010). As a consequence, there has been little success translating the knowledge obtained from model organisms to therapeutically valuable outcomes. This constraint can only be addressed by studying neurodegenerative diseases directly on humans.

The recent increase in the generation of digital data including imaging, genotypic, and phenotypic information coupled with a fast development of computational methods allows us to obtain new insights from this massive amount of data. Major efforts from consortiums such as the Alzheimer’s Disease Initiative (ADNI), the Australian Imaging, Biomarkers & Lifestyle Study of Ageing (AIBL), the Parkinson’s Progression Markers Initiative (PPMI) or more recently the United Kingdom Biobank have made available an unprecedented amount of data, which can be exploited through the use of big data algorithms.

Having large amounts of information coming from heterogeneous populations and acquisition systems increases the power of statistic approaches and reduces the risk of bias. Big data approaches are inherently limited since in general they cannot be used to perform causal inference, but rather to find correlations or associations between variables. However, these correlations have proven to be an invaluable tool for hypothesis generation, drug and safety surveillance, disease and treatment heterogeneity among many other applications (Lee and Yoon, 2017).

Such computational models have been used for example to build a model of senescence signaling via DNA damage, insulin-TOR, FoxO3a transcription factors, oxidative stress response, mitochondrial regulation, and mitophagy using a systems biology approach (Dalle Pezze et al., 2014). A recent review of mathematical models in neurodegenerative diseases reports several mathematical models involving some of the processes we addressed above, such as energy metabolism and synaptic plasticity (Lloret-Villas et al., 2017). A multivariate Bayesian model of biomarker measurements was used to estimate AD dementia onset age (Bilgel and Jedynak, 2019). Similarly, multivariate models have been used to relate image based morphological features to the risk of dementia (Gutierrez Becker et al., 2018; Cole et al., 2019).

Recent trends in stem cell methods have also opened new possibilities for human brain cell cultures, which can in turn be used to model neurodegeneration. Induced pluripotent stem cells and neural stem cell cultures can now be used to generate human neurons from human and sometimes patient cells (Abud et al., 2017; Korhonen et al., 2018). This has been reviewed extensively elsewhere (Xie and Tang, 2016; Robbins and Price, 2017).

## Conclusion

Disease is typically defined in juxtaposition to the state of health, which has been even more generally defined as “a state of complete physical, mental, and social well-being” (International Health Conference, 2002). However, in order to characterize a bodily or mental state as diseased, we need a more practical, operational definition of the healthy state, characterized in terms of physiological parameters and defined by the description of the steady state functioning of the body and its subsystems.

Typically, pathophysiological events of neurodegeneration are detected through behavioral observations and self-reporting and subsequently confirmed and classified using neurological examination and diagnostic imaging. The result of this diagnostic approach usually reveals typical defining features for each neurological disease (such as hemorrhage, tumor, or deformations), that allows the classification of the disorder. However, these defining characteristics are not necessarily the root causes of the disease but rather elements of the disease manifestation or even merely epiphenomena. In addition to the complex and usually obscure causal relationships giving rise to brain disorders, a parameter that further complicates the identification of causation is the widespread and intricate comorbidities between presumably distinct and unrelated disorders.

Together, these facts limit the therapeutic potential to treat the symptoms rather than the causes. The complex causality chain hinders the early detection and treatment of the root causes of brain disorders overall and neurodegeneration more specifically. These limitations highlight the critical need for a drastic shift of perspective and the improvement of the models used to investigate and understand neurodegenerative diseases.

Given the complexity of the brain and its disorders, researchers and clinicians have retreated to a highly segregated approach, focusing on describing subsets of the symptoms of each disease and trying to identify mechanistic explanations and treatments for those symptoms. However, the lack of a holistic perspective, taking into account the deviations from the default homeostatic state at an organismal level, renders this endeavor nearly impossible and doomed to fail.

Importantly, characterizing the expected, default healthy brain, is a necessary precondition to being able to identify and meaningfully interpret the deviations from it. Thus, characterizing and understanding the brain function in health is of paramount importance in the attempt to understand and treat the diseased brain.

In summary, there is an identifiable need for an interdisciplinary approach to explore the brain function at multiple levels, from molecular to systems, while maintaining a holistic perspective that treats the brain as an element of a complex system, rather than a separate entity. Using this approach, fundamental research can proceed hand-in-hand and maintain a constant dialogue with clinical research, to propose, improve, and revise current models of health and disease.

We hope that our work inspires the opening of new research avenues among those interested in understanding and pursuing the complexities of the “neuro-degenerating” brain focusing on lifespan processes. Therefore, the proposed etiological paths and suggestions will be important guidelines for future cross-discipline research to overcome the translational roadblock and to develop causative treatments for neurological disorders.

## Author Contributions

AP, GG-O, and MZ applied for funding and organized the workshop. AK, AP, AV, BG-B, MZ, NK, and XC were participants of the subgroup “The Vascular Origin of Neurodegeneration.” AV-C, JP-V, KR, KW, LR, and SC-O of the subgroup “Cellular Senescence as the Origin of Neurodegeneration.” CP-C, EM-M, GG-O, HG, and JL-N of the subgroup “Body–Brain Trophic Interactions as the Origin of Neurodegeneration.” Accordingly, the authors wrote the respective sections of this manuscript, except CP-C who together with TS wrote the section “Gut Dysbiosis as the Origin of Neurodegeneration” with contributions from KW. AP contributed to the section “Body–Brain Trophic Interactions as the Origin of Neurodegeneration.” BG-B wrote the section on “Studying Human Disease in Human Systems.” AK on “The Lack of Adequate Experimental Models to Study the Etiology.” NK wrote the conclusion. MZ wrote the remaining sections of the manuscript. AV commented on the manuscript. All authors discussed and commented on the final version of the manuscript.

## Conflict of Interest Statement

The authors declare that the research was conducted in the absence of any commercial or financial relationships that could be construed as a potential conflict of interest.
